# An investigation of the relationship between psychopathy and greater gray matter density in lateral prefrontal cortex

**DOI:** 10.1017/pen.2019.8

**Published:** 2019-10-18

**Authors:** Emily N. Lasko, David S. Chester, Alexandra M. Martelli, Samuel J. West, C. Nathan DeWall

**Affiliations:** 1 Department of Psychology, Virginia Commonwealth University, Richmond, VA, USA; 2 Department of Psychology, University of Kentucky, Lexington, KY, USA

**Keywords:** psychopathy, ventrolateral prefrontal cortex, gray matter density, voxel-based morphometry

## Abstract

Psychopathic traits predispose individuals toward antisocial behavior. Such antagonistic acts often result in “unsuccessful” outcomes such as incarceration. What mechanisms allow some people with relatively high levels of psychopathic traits to live “successful”, unincarcerated lives, in spite of their antisocial tendencies? Using neuroimaging, we investigated the possibility that “successful” psychopathic individuals exhibited greater development of neural structures that promote “successful” self-regulation, focusing on the ventrolateral prefrontal cortex (VLPFC). Across two structural magnetic resonance imaging studies of “successful” participants (Study 1: *N* = 80 individuals in long-term romantic relationships; Study 2: *N* = 64 undergraduates), we observed that gray matter density in the left and right VLPFC was positively associated with psychopathic traits. These preliminary results support a compensatory model of psychopathy, in which “successful” psychopathic individuals develop inhibitory mechanisms to compensate for their antisocial tendencies. Traditional models of psychopathy that emphasize deficits may be aided by such compensatory models that identify surfeits in neural and psychological processes.

Some people have the keen ability to con, manipulate, and harm others, all while avoiding the scrutiny of their peers or the public eye. The mechanisms that differentiate these “successful” psychopaths from others who have similar sinister impulses but are unable to remain in civilized society remain incompletely understood. Traditionally, psychopathy is defined by its deficiencies (e.g., impaired empathy; Blair, 2005; Hare, 2003). However, fewer investigations have examined the potential psychological and neural advantages that support the “successful” side of the psychopathy spectrum. The present study sought to identify neurobiological mechanisms that help explain why some individuals high in psychopathy are able to lead “successful” lives.

## 1. Psychopathy: Definitions and distinctions

Psychopathy is mainly characterized by shallow affect, callousness, impulsivity, lack of empathy, and criminal tendencies (Hare, 2003; Lykken, 1995). Although the term psychopathy will typically bring to mind people who commit horrendous acts of violence, this actually describes only a small subset of the wide range of psychopathy manifestations. These psychopathic traits can be distilled into two subtypes that demonstrate different behavioral and psychophysiological profiles: primary and secondary psychopathy (Gao & Raine, 2010; Karpman, 1948; Skeem, Johansson, Andershed, Kerr, & Louden, 2007; Skeem, Poythress, Edens, Lilienfeld, & Cale, 2003). *Primary psychopathy* is characterized by antagonism, callous affect, grandiosity, manipulative behavior, selfishness, low anxiety, and a charismatic interpersonal style (Karpman, 1948; Lykken, 1995; Miller, Gaughan, & Pryor, 2008; Zeier, Maxwell, & Newman, 2009). Individuals higher in primary psychopathy have an increased inclination toward instrumental or relational aggression, as opposed to reactive, and often show intact or increased executive functioning and cognitive empathy (Blair, 2005; Gao & Raine, 2010; White, Gordon, & Guerra, 2015). There is a degree of overlap between primary and secondary subtypes, such as the lack of emotional empathy and similar antisocial tendencies (Blair, 2005; Lykken, 1995; Skeem et al., 2007). However, when examined in isolation the manifestation of secondary psychopathy paints a much different picture than that of the cold, calculating primary psychopathy subtype.

*Secondary psychopathy* more closely resembles the impulsive, neurotic, and antisocial lay conceptualization of a “psychopath” (Karpman, 1948; Kimonis, Frick, Cauffman, Goldweber, & Skeem, 2012; Miller et al., 2008; Skeem et al., 2003, 2007). In stark contrast to individuals higher in primary traits, people high in secondary psychopathy tend to show more of the behavioral and antisocial characteristics such as deviant behavior, impulsivity, and reactive aggression (Falkenbach, Poythress, & Creevy, 2008; Karpman, 1948; Skeem et al., 2003, 2007). Decades of research has tended to focus on a deficit model of psychopathy, to which we turn next.

## 2. Deficit approaches to psychopathy

Primary and secondary psychopathy are often conflated as one construct known for deficiencies in psychological (e.g., behavioral inhibition) and neurological (e.g., cortical thickness) characteristics that are robustly supported by psychological, physiological, and neurological evidence (Lykken, 1995; Shirtcliff et al., 2009; Yang, Raine, Colletti, Toga, & Narr, 2009). Across population types, from offenders to students, research has consistently shown *deficits* in several domains of functioning, primarily related to secondary psychopathy (Blair, 2005; Decety, Skelly, & Kiehl, 2013; Johnson et al., 2014; Welker, Lozoya, Campbell, Neumann, & Carré, 2014). Compared to primary psychopathy, individuals higher in secondary psychopathy have shown deficits in both emotional and cognitive empathy, poor interpersonal functioning, more symptoms of severe mental illness, decreased executive functioning, and impaired information processing abilities (Blair, 2005; Gao & Raine, 2010; Skeem et al., 2007).They have also shown abnormal amygdala and prefrontal cortex (PFC) function (Brook & Kosson, 2013) – areas of the brain related to both emotion and behavioral control, respectively.

Furthermore, structural and functional magnetic resonance imaging (MRI) studies of incarcerated males have shown negative associations between overall psychopathy and gray matter volume in paralimbic regions, areas largely involved in emotion and empathy, including the amygdala, insula, hippocampus, temporal pole, and orbitofrontal cortex (OFC; Ermer, Cope, Calhoun, Nyalakanti, & Kiehl, 2012; Ermer, Cope, Nyalakanti, Calhoun, & Kiehl, 2013). Similarly, both gray matter volume and concentration in temporal and paralimbic regions were significantly decreased in a group of male offenders high in overall psychopathy, which accounted for about 20% of the variance in overall psychopathy scores (Ermer et al., 2012). This finding has been fairly consistent across similar studies (Cope et al., 2012; Müller et al., 2008; Pera-Guardiola et al., 2016). Evidence has continued to accumulate linking overall psychopathy to decreases in volume and/or function in areas of the brain related to emotion and behavioral control (Cope et al., 2012; Tiihonen et al., 2008). Specifically, offenders high in both primary and secondary psychopathy, compared to offenders on the low end of the spectra, showed deficient affect-related activation within limbic regions and the left inferior frontal gyrus when asked to recall negatively valenced words (Kiehl et al., 2001). Similarly, offenders high in overall psychopathy showed decreased functional connectivity between prefrontal regions and the amygdala in a facial emotion matching task (Contreras-Rodríguez et al., 2014).

Together, the evidence suggests a decreased ability to inhibit reactions to strong emotions as well as inhibited overall reactivity to those emotions. In further support of these conclusions, violent offenders who met the clinical criteria for psychopathy, compared to those who did not meet the criteria, exhibited significantly decreased gray matter volume within temporal regions and the anterior medial PFC (Gregory et al., 2012). These regions of the brain are particularly involved in empathy and guilt – attributes that psychopaths notoriously lack. These psychopathy-linked deficits are crucial to our understanding of this construct, yet these findings did not separate psychopathy into its primary and secondary subtypes. More importantly, the focus on forensic and clinical samples ignores the vast number of individuals with psychopathic traits, who have “successfully” integrated into society. These “successful” psychopaths may not show such deficits.

## 3. Neural and psychological bases of “successful” psychopathy

Despite the wealth of literature on psychopathy in incarcerated populations, there is also considerable evidence suggesting that primary and secondary psychopathic traits exist along a spectrum throughout the general population (Falkenbach et al., 2008; Miller, Maples-Keller, & Lynam, 2016; Neumann & Hare, 2008; Patton, Smith, & Lilienfeld, 2017; Smith, Watts, & Lilienfeld, 2014). Reflecting the continuous nature of the construct, psychopathy has been mapped onto the Five-Factor Model of personality, the “gold standard” measurement of stable dispositional personality tendencies (Lynam & Widiger, 2007; Miller & Lynam, 2003; Miller, Lynam, Widiger, & Leukefeld, 2001). Given that some individuals who possess psychopathic tendencies can adapt and thrive in society (e.g., attend college, maintain steady and lucrative careers), then our understanding of psychopathy should be comprised of more than mere deficits. In fact, certain abilities and traits appear to remain intact for some individuals high in psychopathy, particularly for those higher in primary psychopathic traits. Therefore, it is possible that there is some compensatory feature that allows some individuals with more psychopathic tendencies to override their antisocial impulses.

“Successful” psychopathy has been defined in several ways but essentially identifies manifestations of psychopathy in which its most adaptive traits, such as low anxiety and fearlessness, are featured most prominently (Lilienfeld, Watts, & Smith, 2015; Smith et al., 2014). The adaptive potential of certain psychopathic traits becomes most apparent when examined in non-forensic or noninstitutionalized populations. Studies with samples taken from temporary employment agencies have shown that those higher in “successful” psychopathy (i.e., no history of arrests/convictions) exhibited enhanced executive function over and above that of individuals low in overall psychopathic traits (Gao & Raine, 2010). Granted, “successful” psychopathy cannot be held equivalent to primary psychopathy. However, individuals in noninstitutionalized populations known to be relatively “successful” (e.g., business majors) tend to score significantly higher in primary psychopathy compared to the general population but score similarly to the general population in secondary psychopathy (Wilson & McCarthy, 2011). It is yet unclear what biological mechanism might facilitate these individuals’ achievements.

## 4. The VLPFC: A hub for self-regulation

The ventrolateral prefrontal cortex (VLPFC) may be one brain mechanism responsible for the more “successful” variants of psychopathy. Comprising the inferior frontal gyrus, the VLPFC has often been implicated in the regulation of aggressive and impulsive behavior and serves a more general role in behavioral inhibition processes and regulating emotions (Coccaro, Sripada, Yanowitch, & Phan, 2011; Pawliczek et al., 2013; Raine et al., 1998). The VLPFC is particularly implicated in emotion regulation via adaptive tuning of the amygdala, an area robustly associated with the generation of negative emotion (Blair, 2007; Larson et al., 2013). Indeed, VLPFC activation often exhibits an *inverse* relationship with amygdala activity during the active suppression of negative emotions (Phan et al., 2005). Although the VLPFC plays a key role in the regulation of negative emotion and impulsive behaviors, both of which are central to the general construct of psychopathy, there has been little evidence to suggest that deficits or irregularities in this region are associated with psychopathy (Yang & Raine, 2009). Further, psychopathy is not linked to any structural deficits within the VLPFC (Boccardi et al., 2010; Ermer et al., 2012, 2013; Yang, & Raine, 2009). Given the lack of deficits in this region and its crucial role in promoting the psychological processes that “successful” psychopathic individuals would need, we expect that the VLPFC should be intact, or even potentially enhanced, in such individuals. Finally, such VLPFC surfeits among psychopathic individuals should explain, in part, their ability to inhibit antisocial behavior.

## 5. Study 1

Research has yet to empirically examine the hypothesis that psychopathic individuals within “successful” populations will exhibit *greater* VLPFC gray matter density than their less psychopathic counterparts. In Study 1, we sought to test this hypothesis in a sample of adults in long-term romantic relationships. This inclusion criterion served as an adequate indicator of “successful” psychopathy as these individuals have inhibited antisocial behaviors well enough to have sustained such relationship.

## 6. Methods

### 6.1 Participants

Participants were 80 right-handed, healthy adults (51% female; age: *M* = 21.61, SD = 3.73, range = 18–35) recruited from an introductory psychology subject pool (*n* = 22) and the surrounding Richmond, Virginia community (*n* = 58). Each participant was screened as a member of a romantic couple that was opposite-sex, heterosexual, and exclusively monogamous for at least six continuous months. All participants received $100 (community members) or course credit (undergraduates) and an image of their brain in exchange for their participation. Participants were screened via an online self-report questionnaire for existing major medical conditions, developmental disorders, body mass index above 30, claustrophobia, mental or neural pathology, metallic objects in the body, prior head trauma, or current psychoactive medication use. Those who reported any of these conditions were excluded from the study.

### 6.2 Materials

#### 6.2.1 Short Dark Triad 3

The Short Dark Triad (SD3; Jones & Paulhus, 2014) is a 27-item brief measure of the Dark Triad of personality traits. The three 9-item subscales individually assess trait psychopathy (e.g., “it’s true that I can be mean to others”), narcissism (e.g., “I like to get acquainted with important people”), and Machiavellianism (e.g., “it’s not wise to tell your secrets”). Items are rated along a Likert scale ranging from 1 (*strongly disagree*) to 5 (*strongly agree*). The SD3 does not separately quantify the primary and secondary facets of psychopathy. However, the psychopathy subscale of the SD3 shows stronger correlations with primary psychopathy than secondary psychopathy (Jones & Paulhus, 2014).

### 6.3 Procedure

Participant couples arrived at Virginia Commonwealth University’s Collaborative Advanced Research Imaging center, where they had the study explained to them and were again screened to ensure they would be safe and comfortable in the MRI environment. Participants were then placed, one at a time, in an MRI scanner and had a high-resolution structural scan taken of their brain. After a series of unrelated structural and functional scans, participants exited the scanner and completed a battery of questionnaires including SD3 and a demographics survey. Finally, all participants were debriefed and dismissed.

### 6.4 MRI data acquisition and analyses

All MRI data were obtained using a 3.0T Philips Ingenia scanner. Structural images were acquired using a T1-weighted MPRAGE scan: 1 mm^3^ isotropic voxel size, echo time (TE): 3.7 ms, repetition time (TR): 8.1 ms, flip angle: 6°, field-of-view (FOV): 240 × 259 mm, matrix size: 240 × 256 mm, 160 sagittal slices.

**Figure 1.**
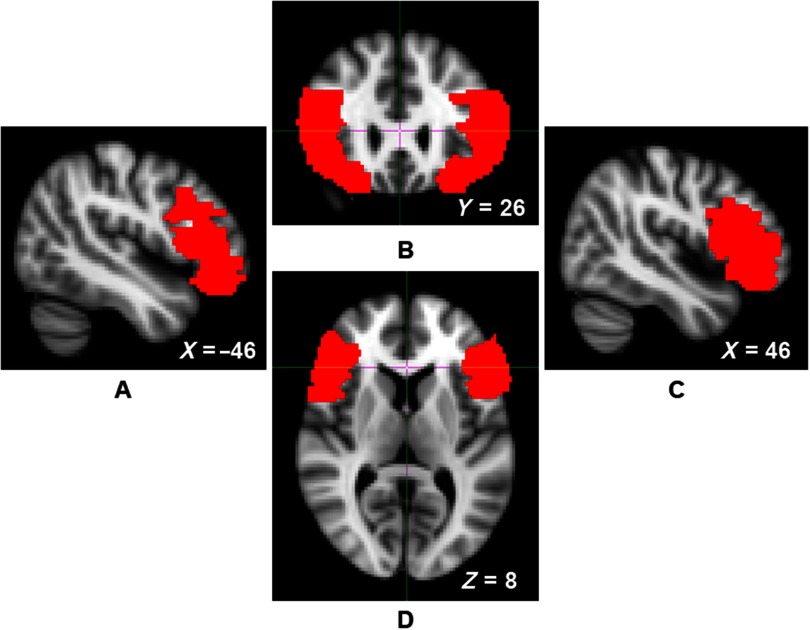
Left sagittal (panel A), coronal (panel B), right sagittal (panel C), and axial (panel D) views of the ventrolateral prefrontal cortex (VLPFC) mask, indicated by red voxels.

The Oxford Centre for Functional MRI of the Brain’s Software Library (FSL version 5.0) was used to conduct all pre-processing and voxel-based morphometry analyses (Smith et al., 2004; Woolrich et al., 2009). Structural data were analyzed with FSL-VBM (Douaud et al., 2007; http://fsl.fmrib.ox.ac.uk/fsl/fslwiki/FSLVBM), an optimized VBM protocol (Good et al., 2001). First, structural images were brain-extracted and gray matter-segmented before being registered to the Montreal Neurological Institute (MNI 152) standard space using nonlinear registration (Andersson, Jenkinson, & Smith, 2007). The resulting images were averaged and flipped along the x-axis to create a left-right symmetric, study-specific gray matter template. Second, all native gray matter images were nonlinearly registered to this study-specific template and modulated to correct for local expansion or contraction due to the nonlinear component of the spatial transformation. The modulated gray matter images were then smoothed with an isotropic Gaussian kernel (*σ* = 3.5 mm). Gray matter estimates were then extracted from this study-specific template to create density estimates for the left and right VLPFC of each participant. VLPFC masks were defined using the Automated Anatomical Labeling atlas mask for the opercular, triangular, and orbital aspects of the inferior frontal gyrus (Figure 1; Tzourio-Mazoyer et al., 2002).

## 7. Results

### 7.1 Descriptive statistics

Psychopathy scores demonstrated substantial between-subject variability but inadequate internal consistency (Table 1).

**Table 1.** Descriptive statistics for Short Dark Triad (SD3) scores (Study 1)

Measure	*M*	SD	Observed range	Cronbach’s *α*
SD3	1.81	0.55	1.00–3.22	.57

### 7.2 Correlations

**Figure 2.**
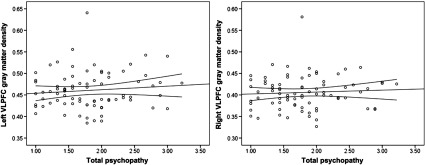
Scatterplots depicting the zero-order correlations (Study 1) between total psychopathy and left ventrolateral prefrontal cortex (VLPFC) gray matter density (left panel) and between total psychopathy and right VLPFC gray matter density (right panel).

Psychopathy was not significantly associated with left or right VLPFC gray matter density (Table 2; Figure 2). Controlling for gender (1 = male, −1 = female), the associations between left, *r*(78) = .15, *p* = .195, and right, *r*(78) = .17, *p* = .128, VLPFC density and psychopathy did not reach statistical significance. Gender was not associated with right or left VLPFC gray matter, but was positively associated with psychopathy (Table 2).

**Table 2.** Zero-order correlation coefficients and associated p values (in parentheses) for Study 1

	1	2	3
SD3 Psychopathy	–	–	–
Left VLPFC	.10 (.359)	–	–
Right VLPFC	.06 (.576)	.82 (<.001)	–
Gender	.44 (<.001)	−.07 (<.001)	−.21 (.070)

*Note*. Gender was coded: 1 = males, −1 = females. SD3 = Short Dark Triad, VLPFC = ventrolateral prefrontal cortex.

## 8. Study 2

Study 2 sought to address a fundamental limitation of Study 1 (i.e., using overall psychopathy scores, as opposed to differentiating between primary and secondary psychopathy facets). We accomplished this goal by examining the VLPFC gray matter density of another “successful” population (i.e., undergraduate students) alongside their levels of primary and secondary psychopathy. Based on evidence suggesting the adaptive potential of *primary* psychopathy traits (Anestis, Harrop, Green, & Anestis, 2017; Lilienfeld et al., 2015; Patton et al., 2017; Smith et al., 2014), we hypothesized that *primary*, and not secondary, psychopathy traits would be positively associated with VLPFC gray matter density.

## 9. Methods

### 9.1 Participants

Participants were 64 right-handed, healthy undergraduate students^1^ (53.1% female; age: *M* = 18.76, SD = 0.98, range = 18–22) recruited from an introductory psychology participant pool. All participants received course credit and an image of their brain in exchange for their participation. The current sample was obtained by combining data from two existing MRI datasets from two separate studies with identical MRI acquisition parameters. Participants were screened via an online self-report questionnaire for MRI safety and comfort criteria identical to Study 1.

### 9.2 Materials

#### 9.2.1 Levenson’s self-report psychopathy scale

Participants were assessed for psychopathic traits and tendencies using Levenson’s Self-Report Psychopathy Scale (LSRP; Levenson, Kiehl, & Fitzpatrick, 1995). The 26-item measure was developed based on the Psychopathy Checklist (PCL-R; Hare & Neumann, 2006) for use in community and student populations to measure both primary (16 items; e.g., “I enjoy manipulating other people’s feelings”) and secondary psychopathy (10 items; e.g., “I quickly lose interest in the tasks I start”). Participants rate statements about their personality on a Likert scale ranging from 1 (strongly disagree) to 4 (strongly agree).

### 9.3 Procedure

Participants arrived at the laboratory where they completed a battery of questionnaires including the LSRP and a demographics survey. Several days after the survey session, participants arrived at the University of Kentucky’s Magnetic Resonance Imaging and Spectroscopy Center where they were again screened to ensure they would be safe and comfortable in the MRI environment. Participants were then placed in an MRI scanner and had a high-resolution structural scan taken of their brain. Participants then exited the scanner and after a series of unrelated tasks, they were debriefed and dismissed.

### 9.4 MRI data acquisition and analysis

All MRI data were obtained using a 3.0 Tesla Siemens Magnetom Trio scanner. Structural images consisted of a T1-weighted MP-RAGE scan: 1 mm^3^ isotropic voxel size, TE: 2.56 ms, TR: 1.69 s, flip angle: 12°, FOV: 224 × 256 mm, matrix size: 256 × 256, 176 sagittal slices. The VBM analysis and extraction procedure was identical to Study 1.

## 10. Results

### 10.1 Descriptive statistics

Primary and secondary psychopathy scores demonstrated sufficient internal consistency and substantial between-subject variability (Table 3). The range of primary and secondary psychopathy scores obtained in the present sample (Table 3) was consistent with previous studies that had similar sample demographics (Prado, Treeby, & Crowe, 2015; Tsang, Salekin, Coffey, & Cox, 2017).

**Table 3.** Descriptive statistics for the Levenson Self-Report Psychopathy Scale (LSRP) subscale scores (Study 2)

Measure	*M*	SD	Observed range	Cronbach’s *α*
LSRP – Primary	2.04	0.50	1.06–3.75	.80
LSRP – Secondary	1.84	0.43	1.20–2.90	.62

### 10.2 Correlations among psychopathy and VLPFC gray matter density

**Figure 3.**
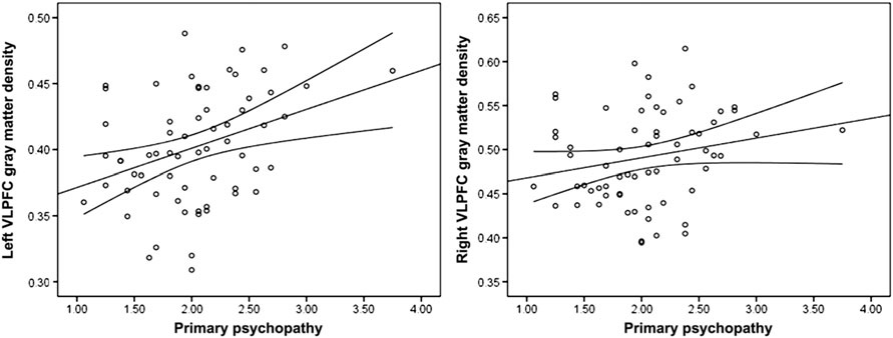
Scatterplots depicting the zero-order correlations (Study 2) between primary psychopathy and left ventrolateral prefrontal cortex (VLPFC) gray matter density (left panel) and between primary psychopathy and right VLPFC gray matter density (right panel).

Zero-order correlations are presented in Table 4. Primary psychopathy was positively associated with secondary psychopathy, left VLPFC gray matter density, and marginally associated with right VLPFC gray matter density (Figure 3; Table 4). Supporting the specificity of our predictions to primary psychopathy, secondary psychopathy was not associated with left or right VLPFC gray matter density. The associations between primary psychopathy and left and right VLPFC gray matter density remained similar while controlling for secondary psychopathy, *r*(62) = .30, *p* = .017, and *r*(62) = .18, *p* = .153, respectively. Controlling for gender, the association between primary psychopathy and both left and right VLPFC density increased in strength, *r*(62) = .40, *p* = .002, and *r*(62) = .27, *p* = .031, respectively. Secondary psychopathy remained unassociated with left and right VLPFC gray matter density, *r*(62) = .21, *p* = .100, and *r*(62) = .12, *p* = .369, respectively.

**Table 4.** Zero-order correlation coefficients and associated *p* values (in parentheses) for Study 2 variables

	1	2	3	4	5
1. LSRP – Primary	–	–	–	–	–
2. LSRP – Secondary	.34 (.006)	–	–	–	–
3. Left VLPFC	.35 (.005)	.22 (.081)	–	–	–
4. Right VLPFC	.22 (.088)	.13 (.294)	.87 (<.001)	–	–
5. Gender	.21 (.098)	−.10 (.442)	−.14 (.266)	−.22 (.083)	–

*Note*. Gender was coded: 1 = male, −1 = female. LSRP = Levenson’s Self-Report Psychopathy Scale, VLPFC = ventrolateral prefrontal cortex.

## 11. Internal meta-analysis

**Figure 4.**
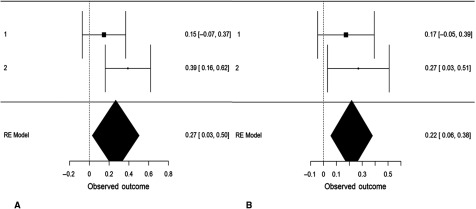
Forest plots depicting an internal random-effects meta-analysis on the correlations between psychopathy and left ventrolateral prefrontal cortex (VLPFC) gray matter density (panel A) and between psychopathy and right VLPFC gray matter density (panel B), across both studies. Values on the left of each panel represent the study of origin for each effect and values on the right of each panel represent individual effect sizes and their associated 95% confidence intervals. Values in the bottom-right of each panel indicate meta-analytic effect size estimates and their associated 95% confidence intervals. Study 2 values reflect correlations between primary psychopathy and VLPFC gray matter density.

**Figure 5.**
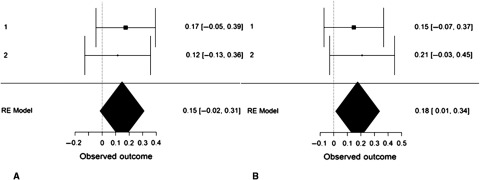
Forest plots depicting an internal random-effects meta-analysis on the correlations between psychopathy and left ventrolateral prefrontal cortex (VLPFC) gray matter density (panel A) and between psychopathy and right VLPFC gray matter density (panel B), across both studies. Values on the left of each panel represent the study of origin for each effect and values on the right of each panel represent individual effect sizes and their associated 95% confidence intervals. Values in the bottom-right of each panel indicate meta-analytic effect size estimates and their associated 95% confidence intervals. Study 2 values reflect the correlations between secondary psychopathy and VLPFC gray matter density.

We performed an internal, random-effects meta-analysis across the two studies’ partial correlations (controlling for gender) between SD3 psychopathy (Study 1), LSRP primary psychopathy (Study 2), and left/right VLPFC gray matter density using JASP v.9.0. Using restricted maximum likelihood estimation, we observed a modest correlation between psychopathy and left VLPFC gray matter density, *r* = .27, *SE* = .12, 95% CI = [0.03, 0.50], *Z* = 2.20, *p* = .028 (Figure 4). We also observed a modest correlation between psychopathy and right VLPFC gray matter density, *r* = .22, *SE* = .08, 95% CI = [0.06, 0.38], *Z* = 2.64, *p* = .008. Both of these effects exhibited significant heterogeneity, *Q*(1) = 4.86, *p* = .028 and *Q*(1) = 6.98, *p* = .008, respectively. To ascertain whether these effects were specific to primary psychopathy, rather than secondary, we conducted the meta-analysis a second time using the two studies’ partial correlations (controlling for gender) between SD3 psychopathy (Study 1), LSRP *secondary* psychopathy (Study 2), and left/right VLPFC gray matter density. The correlation between psychopathy and left VLPFC gray matter density remained significant yet was smaller in magnitude, *r* = .18, *SE* = .08, 95% CI [.01, .34], *Z* = 2.11, *p* = .035. The correlation between psychopathy and right VLPFC gray matter density, however, was not significant, *r* = .15, *SE* = .08, 95% CI [−.02, .31], *Z* = 1.77, *p* = .077 (Figure 5).

## 12. Discussion

Lay conceptualizations of psychopathy are often characterized by maladaptive traits (e.g., impulsive violence). Yet some individuals high in psychopathic traits are often very “successful” in life and able to control their antisocial impulses. The present study targeted the neural mechanisms that contribute to the establishment of “successful” forms of psychopathy. More specifically, we tested a *compensatory model of psychopathy*. According to this model, individuals higher in primary (but not secondary) psychopathy develop surfeits in inhibitory mechanisms over time in order to compensate for and overcome their antisocial impulses. Drawing from this framework, we predicted that “successful” psychopathic individuals would exhibit neural indicators of advanced self-regulatory capabilities.

### 12.1 A compensatory neural mechanism

Across both studies, our internal meta-analysis revealed a positive association between (largely primary) psychopathy and VLPFC gray matter density in both the left and right hemispheres. The same pattern was not observed for secondary psychopathy (Study 2). The VLPFC subserves self-regulatory abilities such as impulse control (Coccaro et al., 2011; Pawliczek et al., 2013). Thus, these findings provide preliminary support for the proposed compensatory model of psychopathy by demonstrating an inhibitory neural surfeit among “successful” individuals higher in psychopathy. Traditional, deficit-based models of psychopathy have largely ignored the advantageous abilities that psychopaths possess, which may allow them to evade public scrutiny and thrive within society. Gao and Raine’s (2010) neurobiological model of “successful” and “unsuccessful” psychopathy was one of the pioneering models to theorize about the etiology of these divergent manifestations of psychopathy. Our findings fit well within this model’s tenets, which specify that prefrontally mediated self-regulatory functions are enhanced among “successful” individuals high in psychopathy. The fact that our results were replicated across both hemispheres aligns with this model and other neurobiological models of psychopathy that generally demonstrate bilateral neural differences between “successful” and “unsuccessful” populations (Anderson & Kiehl, 2012). Indeed, meta-analyses examining structural and functional studies of antisocial individuals revealed lateralization effects in nearly every region of interest *except* the VLPFC, which showed no abnormalities in the left or right hemisphere (Yang & Raine, 2009). Although these studies simply identified *lack of deficits* rather than *surfeits* within the VLPFC, this may be due to inherent differences between “successful” and “unsuccessful” populations, which we discuss in the following sections.

### 12.2 “Successful” psychopathy

If replicated, our findings lend preliminary neurological support to the more recently developed view of “successful” psychopathy. In this differential-configuration model (Lilienfeld et al., 2015), the difference between “successful” and “unsuccessful” populations lies in the configuration of psychopathic traits phenotypically expressed. For instance, certain traits such as low disinhibition (i.e., impulse control) and elevated boldness (i.e., reactivity to fearful stimuli), from the Triarchic model of psychopathy (Patrick, Fowles, & Krueger, 2009), may play key roles in the presentation of “successful” psychopathy (Lilienfeld et al., 2015). Yet the ultimate source (e.g., parenting, genetics) and developmental trajectory of heightened gray matter density among “successful” populations of psychopathic individuals remain unknown. Longitudinal and genetic studies of these relationships would help untangle the many possible underlying causes for our observed effects.

### 12.3 Inconsistencies with past research

Our findings stand in contrast with those from several other studies, which found links between psychopathy and *decreased* gray matter density in the left PFC, and no associated increases in gray matter (Müller et al., 2008). We also failed to replicate the commonly observed association between total psychopathy traits and amygdala structure and function (Blair, 2007; Larson et al., 2013; Yang, Raine, Colletti, Toga, & Narr, 2010). However, these studies were conducted within forensic populations, whereas the present two studies used ostensibly “successful” populations (i.e., nonincarcerated individuals in long-term relationships and college students, respectively). Therefore, the inconsistencies discussed above may reflect differences between “successful” and “unsuccessful” populations given that forensic and non-forensic samples differ substantially in terms of substance abuse, trauma, and offense history.

We did not observe any association between VLPFC gray matter density and secondary psychopathy in Study 2. The internal meta-analysis also revealed that the association between psychopathy and right VLPFC gray matter density did not reach significance, and the association between psychopathy and left VLPFC gray matter density decreased in magnitude, when secondary psychopathy scores (Study 2) were entered into the model rather than primary psychopathy scores. These results may be due to inherent neural and lifestyle differences (e.g., substance abuse, trauma) between “successful” and “unsuccessful” populations may have also contributed to the lack of associations found between secondary psychopathy (Study 2) and gray matter density in the present studies. Secondary psychopathic traits, not primary, are characterized by impulsive behavior (Anestis, Anestis, & Joiner, 2009; Karpman, 1948). Secondary psychopathic traits are also more often associated with structural deficits in brain regions involved in behavioral control and abnormalities in the function of these areas (Blair, 2007; Contreras-Rodríguez et al., 2015; Gao & Raine, 2010; Kimonis et al., 2012; Pera-Guardiola et al., 2016). However, these studies are typically conducted within forensic or clinical populations with individuals above college age – factors that could potentially influence various brain regions, including the bilateral VLPFC. Future studies should further examine right and left VLPFC structure and function in psychopathy within the context of both “successful” and “unsuccessful” populations.

### 12.4 Limitations and future directions

The limitations of the current studies should not be understated, as there are several. A main limitation is the relatively small sample sizes across both studies. In the future, attempts should be made to replicate these findings with larger sample sizes. Additionally, the correlational nature of the studies limits the conclusions and interpretations that can be derived from the findings. That is, we do not yet know what caused or precipitated the increase in gray matter for people high in psychopathy or if this increase has any direct effects on behavior. Longitudinal and developmental studies should examine brain structure and function over time, in multiple contexts, and subsequent behavior change to gain a better understanding of possible causal relationships at work.

The use of undergraduates and participants involved in a romantic relationship likely restricted our observed range in psychopathy scores. Although these sample populations likely provided us the best approximation of the “successful” psychopathy construct, the results derived from them may not be generalizable to a broader population. Future studies should attempt to replicate the findings in more diverse samples including forensic and community populations.

A third important limitation is that the present studies did not account for boldness (i.e., LSRP and SD3), which provides an impetus for further exploration into how the left and right VLPFC are *uniquely* related to each component of psychopathy. Low disinhibition and boldness tend to be significantly associated with primary psychopathy within “successful” populations (Berg, Lilienfeld, & Sellbom, 2017; Patton et al., 2017). A relatively large body of research has accumulated that suggests a vital role of boldness in the development of “successful” psychopathy (Lilienfeld et al., 2015; Patrick et al., 2009). As such, it is imperative that future studies assess boldness in relation to self-regulation and VLPFC structure.

Finally, the internal reliability of the psychopathy measure used in Study 1 (the SD3) was inadequate, undermining the robustness of these results. It is possible that the lack of internal consistency can be attributed to the fact that the measure collapses across primary and secondary forms of psychopathy, rather than assessing the two factors individually. These fundamental flaws in the SD3 may potentially also partially explain the lack of significant associations found in Study 1 and the significant (yet smaller) association between psychopathy and left VLPFC gray matter density observed in the internal meta-analysis when Study 2 secondary psychopathy scores were entered into the model. Future research should use more psychometrically reliable measures of psychopathy (e.g., LSRP, Psychopathic Personality Inventory) in order to properly assess the differential relationships between facets of psychopathy and VLPFC structure.

## 13. Conclusions

These findings, while correlational and preliminary, add to a growing literature that suggests a much more nuanced perspective of psychopathy than has conventionally been studied. Past work has largely focused on the psychological and neural deficits that predict the “unsuccessful” aspects of psychopathy. The present studies have identified a potential neurological advantage associated with “successful” psychopathic individuals (i.e., greater VLPFC gray matter density). This anatomical surfeit in a known substrate of inhibitory control may explain the decreased antisocial behavior among “successful” psychopathic individuals. These findings paint a picture of a neural mechanism that underlies a compensatory model of psychopathy, in which “successful” psychopathic individuals develop prodigious self-regulatory strengths in order to compensate for their antisocial impulses. Future research should attempt to replicate and extend these findings as they may translate into intervention and prevention programs and could greatly advance a holistic understanding of psychopathy.

## Footnotes

1 These participants’ gray matter density data were presented in prior publications (Chester & DeWall, 2016; Chester, Lynam, Milich, & DeWall, 2017), though never in relation to psychopathy traits.
